# An optimized protocol for coupling oxygen consumption rates with β-oxidation in isolated mitochondria from mouse *soleus*

**DOI:** 10.1016/j.xpro.2021.100735

**Published:** 2021-08-12

**Authors:** Cristina Sánchez-González, Laura Formentini

**Affiliations:** 1 Departamento de Biología Molecular, Centro de Biología Molecular “Severo Ochoa” (CBMSO), Universidad Autónoma de Madrid, c/ Nicolas Cabrera 1, 28049, Madrid, Spain; 2 Centro de Investigación Biomédica en red de Enfermedades Raras (CIBERER), ISCIII, Madrid, Spain; 3 Instituto de Investigación Hospital 12 de Octubre, i+12, Universidad Autónoma de Madrid, Madrid, Spain

**Keywords:** cell biology, cell isolation, cell membrane, cell separation/fractionation, metabolism

## Abstract

Depending on metabolic requirements, skeletal muscle mitochondria integrate O_2_ consumption and ATP production with lipid, glucose, or amino acid metabolism. Free fatty acids (FFAs) are the main source of energy during rest and mild-intensity exercise. We present a detailed protocol for measuring FFA-β-oxidation coupled with O_2_ respiration by a Clark-type electrode in isolated mitochondria from mouse *soleus* oxidative muscle. We optimized the procedure, including buffer composition, protease treatment, and quantifiable parameters (P/O, Phosphate/Oxygen Ratio; OCR, Oxygen Consumption Rate; RCR,Respiration Control Rate; OSR, Oligomycin Sensitive Respiration).

For complete details on the use and execution of this protocol, please refer to Sanchez-Gonzalez et al. (2020).

## Highlights

• Nagarse disaggregates muscle fibers facilitating mitochondrial isolation
• Mitochondrial oxygen consumption is coupled with palmitoyl-carnitine β-oxidation
• Phosphate/oxygen (P/O) and other ratios (OCR, RCR, OSR) may be easily calculated
• This protocol uses isolated mitochondria from *soleus* but can be adapted to any muscle

## Graphical abstract

**Figure:**
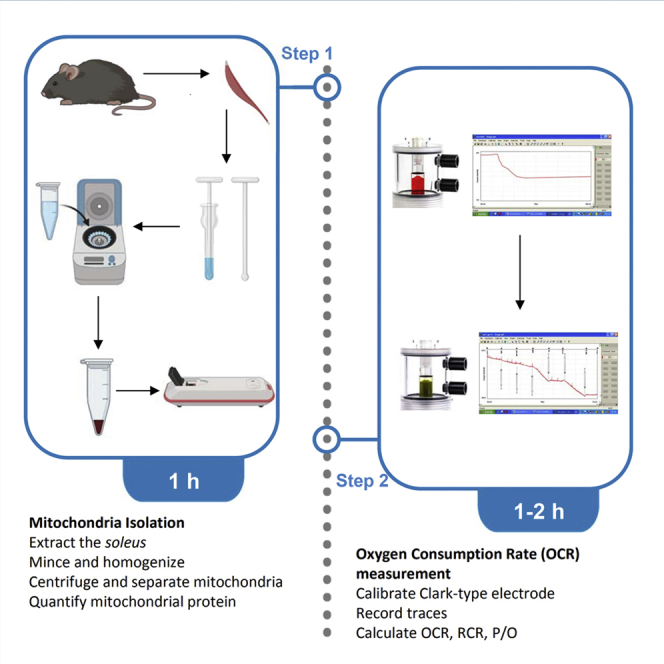

## Teaser

Depending on metabolic requirements, skeletal muscle mitochondria integrate O_2_ consumption and ATP production with lipid, glucose, or amino acid metabolism. Free fatty acids (FFAs) are the main source of energy during rest and mild-intensity exercise. We present a detailed protocol for measuring FFA-β-oxidation coupled with O_2_ respiration by a Clark-type electrode in isolated mitochondria from mouse *soleus* oxidative muscle. We optimized the procedure, including buffer composition, protease treatment, and quantifiable parameters (P/O, OCR, RCR, OSR).

## Before you begin

***Note:*** All animal experiments were approved by the Spanish Animal Experiments Committee (PROEX 183/17) in compliance with the European Community Council Directive Guidelines (EU directive 86/609) and ARRIVE Guidelines. All procedures were performed ensuring minimal discomfort and distress to animals.

### Skeletal muscle mitochondria pre-isolation preparation

**Timing: 30′**

1. Prepare a sufficient amount of extraction (A) and respiration (B) buffers, following the recipe (see below).

**CRITICAL:** A and B buffers can be stored at −20°C for up to 1 month.

2. Thaw buffer A on ice and B at 30°C prior to usage.

**CRITICAL:** Buffer A should be used at 4°C and B at 30°C to ensure proper mitochondria isolation and coupling.

3. Prepare Digestion buffer (C). Weigh Nagarse (0.2 mg/mL in buffer A (see below) to make buffer C; it can be stored at −80°C for up to 6 months with negligible loss of activity).

***Note:*** Nagarse ensures mitochondrial purity, reducing the amounts of endoplasmic vesicles and reticulum (Contreras et al., 2007). Other mild alkaline proteases work similarly. Exact Units of protease/mL should be set up for every muscle.

4. Pre-cool 1× PBS, beakers, tubes, and surgical material for *soleus* extraction.

***Note:*** If possible, work inside a laminar flow cabinet to reduce contaminations during *soleus* dissection.

**CRITICAL:** Use sterilized surgical material by spraying with 70% ethanol (EtOH)

5. Make sure that all of the equipment works well and the centrifuges have been pre-chilled at 4°C.

**CRITICAL:** Make sure that no detergent residues are present on potter and glass material. Detergents may uncouple mitochondria.

6. Prepare a sterilized working space, spraying surgical area with 70% EtOH

### Clark-type electrode preparation

**Timing: 1–1.5 h**

7. Prepare stock solutions for all mitochondria substrates.
  Skm mitochondria may be energized using substrates:
  a. glutamate-malate or any NADH-linked substrates of the NADH:ubiquinone oxidoreductase (allow the entrance of electrons from the ETC-Complex I). Pyruvate-malate are the most commonly used alternative. Final concentration: 10 mM
  b. succinate, natural substrate of the Succinate Dehydrogenase (allow the entrance of electrons from the ETC-Complex II). Final concentration: 10 mM
  c. malate + palmitoyl-carnitine, coupling oxygen consumption with β-oxidation (Nicholls, 2013). Final concentration: 0.5 mM malate; 0.05 mM palmitoyl-carnitine
    **CRITICAL:** Higher concentrations of malate may support NADH-linked electron flow via ETC-complex I.
    ***Note:*** Octanoyl-carnitine or any other FFA-carnitine may be used.
    ***Note:***TMPD/ascorbate may be used as non-natural substrate of the ETC-Complex IV. Final concentration: 2 mM TMPD; 10 mM ascorbate.
    Stocks (50×) are prepared as follows:
  a. 1 M ***malate*** (MW 134.09 g/mol): 134 mg in 1 mL milli-Q H_2_O.
  b. 1 M ***glutamate*** (MW 187.13 g/mol): 187 mg in 1 mL milli-Q H_2_O. Mixed with malate at 1:1 (v:v) to obtain the 500 mM glutamate/malate stock.
  c. 500 mM ***succinate*** (MW 118.09 g/mol): 59.05 mg in 1 mL milli-Q H_2_O.
  d. 50 mM ***malate***: dilute 1:20 the 1 M malate stock with milli-Q H_2_O.
  e. 5 mM ***palmitoyl-carnitine*** (MW 436.07 g/mol): 2.18 mg in in 1 mL milli-Q H_2_O.
  f. 100 mM ***ADP*** (MW 427.2): 42.72 mg in 1 mL milli-Q H_2_O.
  g. 25 mM ***ADP***: dilute 1:4 the 100 mM ADP stock with milli-Q H_2_O.
    **CRITICAL:** Adjust all stock solution pH to 7.4.

8. Prepare stock solutions for all mitochondria inhibitors.
  Skm oxidative phosphorylation (OXPHOS) may be inhibited by using:
  a. Rotenone or piericidin A, inhibitors of the ETC-Complex I (final concentration: 1 μM)
  b. Malonate, inhibitor of the ETC-Complex II (final concentration: 100 μM)
  c. Antimycin A, inhibitor of the ETC-Complex III (final concentration: 1 μM)
  d. Sodium Azide (NaN_3_), inhibitor of the ETC-Complex IV (final concentration 100 mM)
    ***Note:*** KCN (final concentration: 10 μM), can also be used to inhibit ETC-Complex IV. However, it should be noted that pyruvate and high oxygen concentrations may revert KCN inhibition (even at concentrations as high as 1 mM).
  e. Oligomycin, inhibitor of the H^+^-ATP synthase (final concentration: 5 μM)
    Prepare stock solutions for other compounds:
  f. FCCP, an ionophore that uncouples ETC electron flow and O_2_ consumption from ATP production (final concentration: 0.5 μM)
    ***Note:*** FCCP (or CCCP as an alternative) allows the measurement of maximum ETC electron flow capacity that is not limited by the ATP synthase activity (Gnaiger, 2020). FCCP should be titrated in in 0.25–0.5 μM steps until no further increase in oxygen consumption is observed, as excessive FCCP quickly collapses the proton gradient across the inner mitochondrial membrane, leading to a reduction in measured oxygen flux (Brennan et al., 2006).
    Stocks (100×) are prepared as follows:
  a. 100 μM ***rotenone*** (MW 394,41 g/mol): 3.94 mg in 1 mL EtOH to obtain the 10 mM stock. Aliquot and store at −20°C. Dilute 1:100 with milli-Q H_2_O just before use.
  b. 100 μM ***antimycin A*** (MW 534,6 g/mol): 5.34 mg in 1 mL EtOH to obtain the 10 mM stock. Aliquot and store at −20°C. Dilute 1:100 with milli-Q H_2_O just before use.
  c. 500 μM ***oligomycin*** (MW 791 g/mol): 19.8 mg in 1 mL EtOH to obtain the 25 mM stock. Aliquot and store at −20°C. Dilute 1:50 with milli-Q H_2_O just before use.
  d. 500 μM μM ***FCCP*** (MW 254,16 g/mol): 6.35 mg 1 mL EtOH to obtain the 25 mM stock. Aliquot and store at −20°C. Dilute 1:500 with milli-Q H_2_O just before use.
    **CRITICAL:** EtOH stocks may be stored at −20°C up to 1 month. Inhibitor final stocks need to be prepared freshly the day of the experiment and stored on ice, protected from light.
    **CRITICAL:** Make sure to adjust pH to 7.4 in all reagent solutions and maintain all reagents on ice.

9. Set the electrode-bath temperature to 30°C to ensure constant temperature during measurement

***Note:*** Remember that in conditions of O_2_ saturation, at 30°C, oxygen (O) concentration in liquid media is 445 nmol/mL

10. Set up the Clark type electrode (Figure 1) by placing 1 drop of saturated KCl on the top of the platinum electrode and immediately covering it by Teflon membrane, ensuring no bubble is forming.

**Figure 1:**
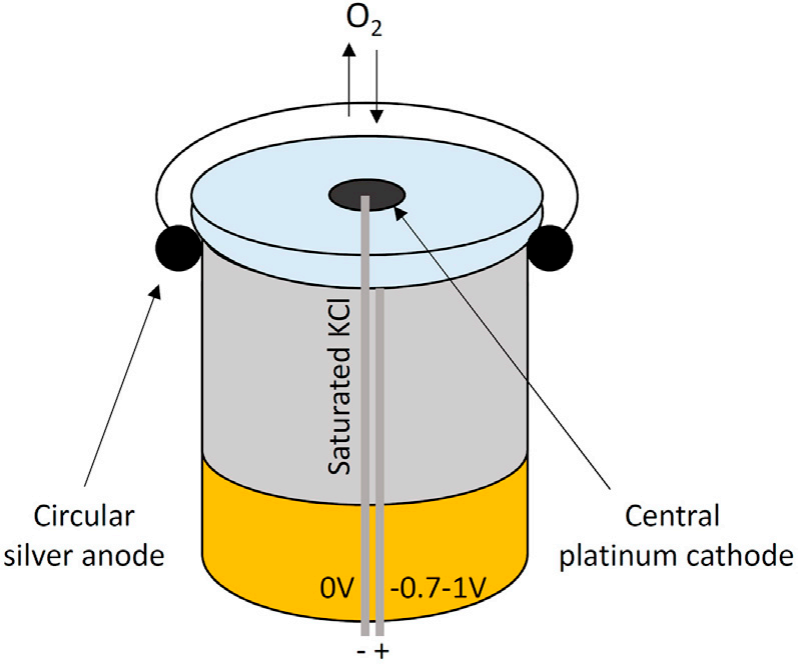
Scheme of the electrode (platinum cathode and silver anode)
11. Immediately place milli-Q water in the electrode working chamber.

**CRITICAL:** Teflon membrane MUST be maintained wet for all the time of the experiment.

12. Turn on the Clark-type electrode (Oxygraph+, Hansatech-instruments, Figure 2).
  a. Turn on the electrode
  b. Turn on the computer
  c. Run “Oxygraph+” (or similar) Software.
  d. Turn on agitation (*Stirrer ON*) to ensure homogenous concentration of O_2_ in the working chamber.

**Figure 2:**
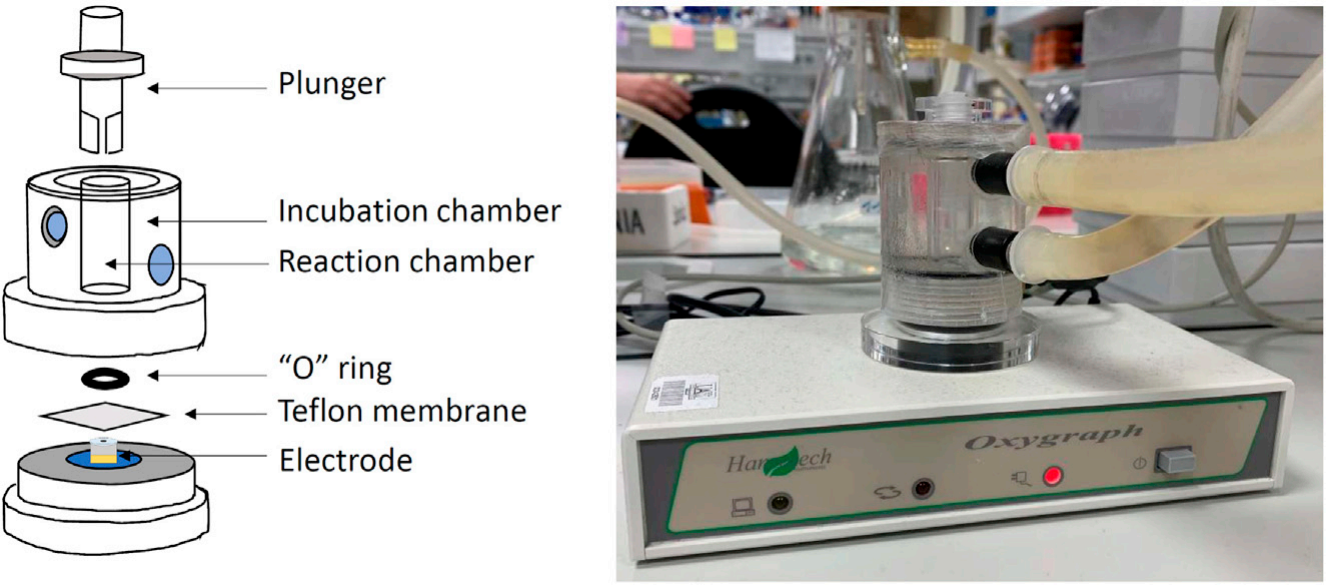
Clark-type electrode Scheme (left) and apparatus (right).
13. Calibrate electrode with dithionite to set 0% oxygen consumption.
  a. Click *Liquid phase calibration*: T 30°C; Stirrer 70 rpms; click *OK* (Figure 3)

**Figure 3:**
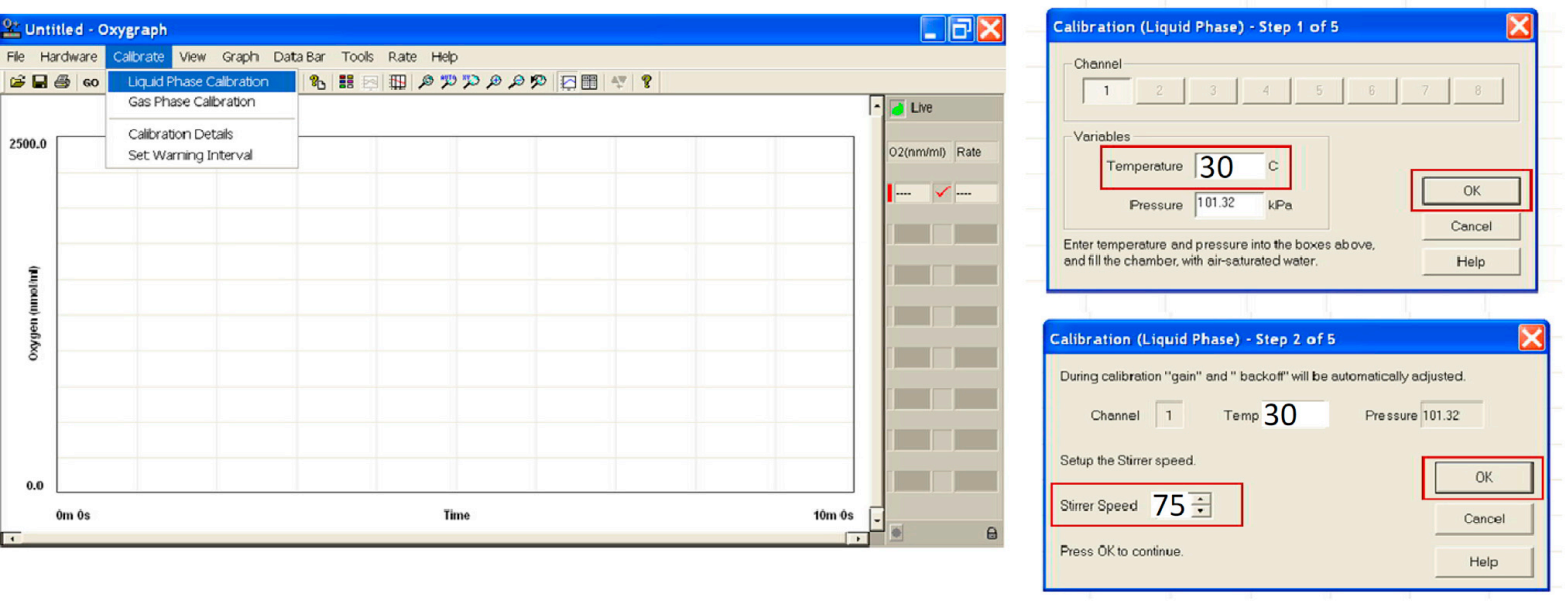
Oxygraph+ calibration interface
  b. Start recording the trace.
  c. Add a pinch of dithionite to set 0% of O_2_. Click *OK.*
  d. The trace will drop until a plateau.
  e. Save new calibration.
14. Wash twice the working chamber with 2 mL milli-Q H_2_O, 2 mL EtOH, 2 mL milli-Q H_2_O, to carefully remove all dithionite residues.

## Key resources table

**Table:** 

REAGENT or RESOURCE	SOURCE	IDENTIFIER
**Antibodies**		

Anti-Calreticulin (dilution 1:1000)	Abcam	Cat#ab92516
Anti-NDUFA9 (dilution 1:1000)	Abcam	Cat#ab14713
Anti-α-Tubulin (dilution 1:1000)	Sigma-Aldrich	Cat#T5168

**Biological samples**		

Healthy oxidative *soleus* muscle	Mus musculus	-

**Chemicals, peptides, and recombinant proteins**		

Sucrose	Sigma-Aldrich	Cat#84100
Tris-HCl	Sigma-Aldrich	Cat#10812846001
EDTA	Sigma-Aldrich	Cat#ED2P
KH2PO4	Sigma-Aldrich	Cat#P0662
K2HPO4	Sigma-Aldrich	Cat#60353
KCl	Merck	Cat#104936
MgCl2	Sigma-Aldrich	Cat#M8266
BSA	Nzytech	Cat#MB04602
EGTA	Sigma-Aldrich	Cat# 324626
Nagarse	Sigma-Aldrich	Cat# P4789 discontinued
Bradford (Bio-Rad Protein Assay)	Bio-Rad	Cat#5000006
Glutamate	Sigma-Aldrich	Cat#49621
Malate	Sigma-Aldrich	Cat#2300
Sodium succinate	Sigma-Aldrich	Cat#S7501
Palmitoyl-carnitine	Sigma-Aldrich	Cat#P1645
TMPD	Sigma-Aldrich	Cat# T7394
Sodium ascorbate	Sigma-Aldrich	Cat#A7631
Rotenone	Sigma-Aldrich	Cat#R8875
Piericidin A	Sigma-Aldrich	Cat# 96861
Malonate	Sigma-Aldrich	Cat# 63409
Antimycin A	Sigma-Aldrich	Cat#A8474
Sodium azide (NaN3)	Sigma-Aldrich	Cat#S2002
KCN	Sigma-Aldrich	Cat# 207810
Oligomycin	Sigma-Aldrich	Cat#O4876
ADP	Sigma-Aldrich	Cat#A2754
FCCP	Sigma-Aldrich	Cat#C2920
Dithionite	Merck	Cat#1065070500
EtOH	Merck	Cat#51976

**Experimental models: Organisms/strains**		

Mus musculus: C57BL/6 6 month-old mice (males)	The Jackson Laboratories	Cat#MGI:5656552

**Software and algorithms**		

Oxygraph+ Software	Hansatech Instruments	-
GraphPad Prism 7	1992-2016 GraphPad Software, Inc	-

**Other**		

Oxygraph+ (Clark-type electrode)	Hansatech Instruments	http://www.hansatech-instruments.com/product/oxygraph-system/
Hamilton syringe (25 and 50 μL)	Sigma-Aldrich	Cat#21492 Cat#24544
KIMBLE Dounce tissue grinder set (Glass to glass homogenizer)	Sigma-Aldrich	Cat#D9063-1SET
Centrifuge	Eppendorf	Cat#5418 R
Moria Iris Forceps	FST	Cat#11370-31
Moria Iris Forceps	FST	Cat#11370-32
Halsted-Mosquito Hemostats	FST	Cat#13008-12
Vannas Spring Scissors	FST	Cat#15000-00
Extra Fine Bonn Scissors	FST	Cat#14084-08
Surgical Scissors	FST	Cat#14001-12
25G needles Sterican 100	Braun	Cat#4657853

## Materials and equipment

### Buffers

**Table:** Extraction Buffer (A)

Reagent	Final concentration	Amount
Sucrose	0.32M	11 g
Tris-HCl	10 mM	121 mg
EDTA 1M	1 mM	100 μL
H_2_O milli-Q	n/a	up to 100 mL
**Total**	**n/a**	**100 mL**

Storage at −20°C until use. Use at 4°C.

**Table:** Respiration Buffer (B)

Reagent	Final concentration	Amount
Sucrose	225 mM	7.7 g
KCl	10 mM	74 mg
MgCl_2_	5 mM	47.6 mg
HK_2_PO_4_	10 mM	134 mg
H_2_KPO_4_	10 mM	30 mg
EGTA	1 mM	38 mg
Tris HCl	10 mM	121 mg
BSA	0.05%	50 mg
H_2_O milli-Q	n/a	up to 100 mL
**Total**	**n/a**	**100 mL**

Storage at −20°C until use. Use at 30°C.

***Optional:*** Nagarse Buffer (C)

**Table:** 

Reagent	Final concentration	Amount
Sucrose	0.32M	11 g
Tris-HCl	10 mM	121 mg
EDTA 1M	1 mM	100 μL
Nagarse	0.2 mg/mL	20 mg
H_2_O milli-Q	n/a	up to 100 mL
**Total**	**n/a**	**100 mL**

Storage at −20°C until use.

***Note:*** A and B buffers were published in (Formentini et al., 2014 )

**CRITICAL:** Adjust pH to 7.4 in all buffers.

## Step-by-step method details

### Skeletal muscle mitochondria isolation

**Timing: [1 h]**

Isolation of *soleus* oxidative skeletal muscle mitochondria by centrifugation steps (Sanchez-Gonzalez et al., 2020).

1. Sacrifice mice in CO_2_ chamber.
2. Extract *soleus* muscles from mice hindlimbs using pre-cooled and sterilized surgical material inside a laminar flow cabinet to reduce impurities.

**Figure 4:**
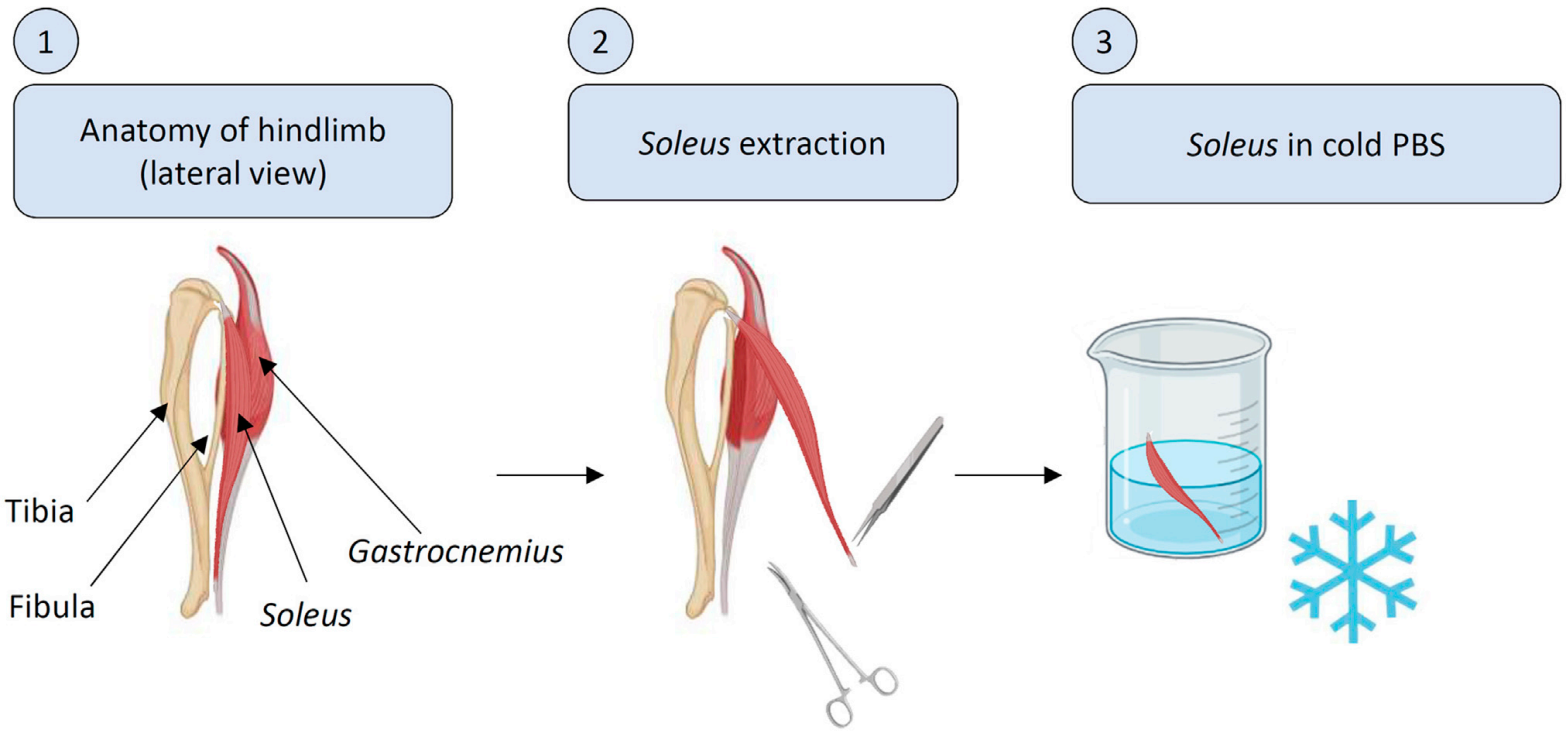
Scheme of *soleus* extraction 1. Lateral view of the anatomy of mouse hindlimb. 2.Extraction of *soleus*: remove fascia, insert fine-tip forceps between distal tendons, liberate Achilles tendon from tibial bone, liberate *soleus* from proximal tendons and extract *soleus*. 3: Immediately transfer *soleus* to cold PBS.

***Note:*** To extract *soleus*, fix the mouse leg in a flexed position to the dissecting table and cover it with cold 1× PBS solution. Remove hair, skin and surrounding fascia. Cut Achilles tendon. Separate the *soleus* muscle from the hindlimb and clean from leftover fascia. (Figure 4, adapted from (Shinin et al., 2009))

**CRITICAL:** Remove as much fascia as possible, as remaining fascia will complicate the isolation of the entire muscle.

***Note:*** To identify *soleus*, pay attention to muscle color. Being deeply oxidative and enriched in mitochondria, *soleus* red color is darker than surrounding muscle. Carefully cut the tendon as close as possible to the knee and separate *soleus*.

**CRITICAL:***Soleus* must be extracted with no white adipose tissue (WAT) deposits to ensure coupling of isolated mitochondria.

3. Weigh *soleus.*

***Note:*** Depending on the age of the animals, 2 or more *solei* are needed to get enough mitochondria for measuring respiration. This also depends on the volume of the chamber and the sensitivity of the Clark-type electrode. This protocol has been optimized for 4 *solei* (a pool of 2 mice/preparation).

***Note:*** Mitochondrial function can be also analyzed *in situ* in permeabilized mouse *soleus* fiber bundles *(**Kuznetsov et al., 2008**)*, allowing 2 respirometer runs per *soleus*, although the method does not allow the measurement of all the parameters reported in this protocol (see below).

**Figure 5:**
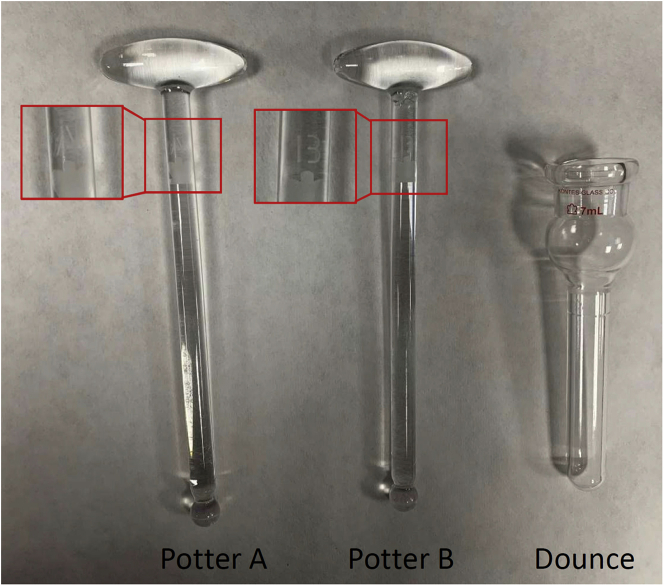
Representative image of potters A and B, and dounce
  d. Immediately transfer the homogenized suspension to previously pre-cooled centrifuge tubes.
  e. Centrifuge 10 min at 700 g at 4°C.
  f. Discard pellet, contains nucleus and intact cells.
  g. Repeat eand f steps once.
    ***Note:*** the double centrifugation is required to improve purity of isolated mitochondria.
  h. Centrifuge supernatant for 10 min at 10,000 g at 4°C.
  i. Eliminate supernatant, and suspend mitochondria enriched pellet in 100 μL of buffer B.
  j. Measure mitochondria protein by Bradford method in a spectrophotometer. A summary for steps a-j is provided in Figure 6.
    ***Note:*** This and other protocols require coupled mitochondria (Nuevo-Tapioles et al., 2020). When coupling is not required (ETC enzymatic activities, blue native, in-gel activities among others protocols (Nuevo-Tapioles et al., 2020)) h and i steps may be repeated once or twice to enhance mitochondrial purity.
    **CRITICAL:** Buffer B may interfere with protein measurement. Ensure to use buffer B for calibration curve.
    **CRITICAL:** Make sure to maintain mitochondria on ice all the time to avoid uncoupling.

4. For isolating mitochondria (on ice). Adapted from (Fernandez-Vizarra et al., 2010):
  a. Wash *soleus* in cold PBS 1× in a pre-cooled 50 mL glass beaker.
  b. Mince muscles in four volumes (depending on muscle weight) of buffer A until pieces are homogeneous with the help of pre-cooled surgical scissors.
    ***Optional:*** Nagarse or other smooth proteases increase mitochondrial purity, reducing the amounts of other organelles. Step b may be performed in buffer C. Incubate 5–10 min on ice to allow Nagarse to act.
    **CRITICAL:** excessive time in Nagarse added buffer may disrupt mitochondria.
    ***Note:*** Mitochondria purity may be verified by WB using specific antibodies against mitochondrial complexes (anti-NDUFA9), cytosolic (anti-tubulin) or other organelle (anti-calreticulin) proteins.
  c. Homogenize in a glass-glass homogenizer (Figure 5):
    - 10 times with potter A (smoother).
    - 15 times with potter B (stronger).
      **CRITICAL:** It is really important not to pass more times with the potters than necessary. Mitochondria may uncouple if the homogenization step is too strong. Exact conditions of homogenization should be optimized for any potter.

**Figure 6:**
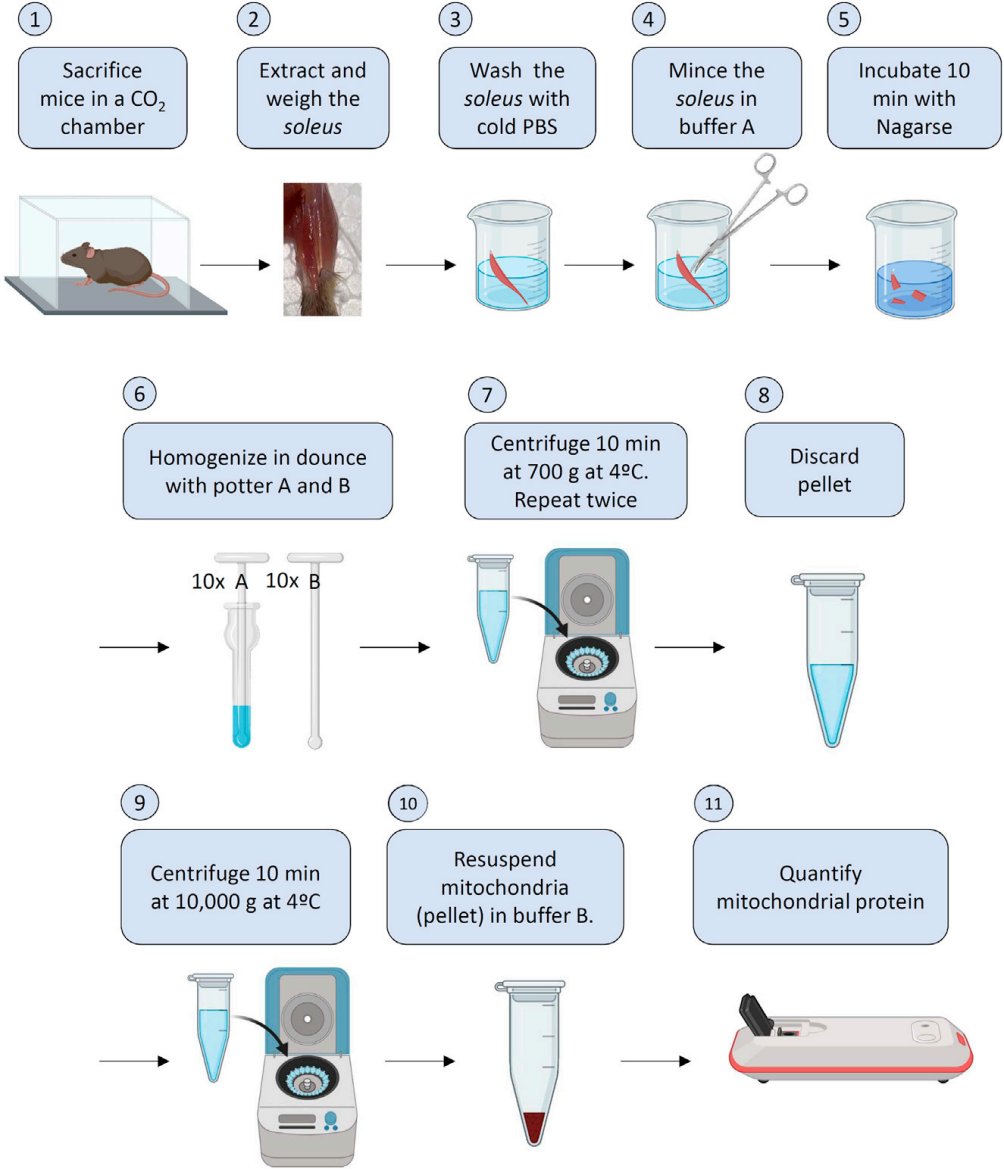
Mitochondria isolation protocol Step1: sacrifice mice in CO_2_ chamber. Step 2: *soleus* extraction and weight. Step 3: *soleus* wash with cold and sterile PBS. Step 4: mince *soleus* in buffer A. Step 5: incubate with Nagarse protease. Step 6: homogenize in glass-glass homogenizer. Step 7: pellet nuclear and membrane rests. Step 8: discard pellet. Step 9: pellet mitochondria. Step 10: resuspend mitochondria in buffer B. Step 11: measure mitochondrial protein.

### OCR measurement by clark-type electrode

**Timing: 1–2 h**

Measurement of oxygen consumption rate (OCR) in isolated mitochondria from mouse *soleus* in a Clark-type electrode (Sanchez-Gonzalez et al., 2020).

5. Add 500 μL of respiration buffer to the electrode working chamber.

***Note:*** the electrode chamber volume may vary between Clark-type electrodes and proper volume should be calibrated to each individual instrument.

6. Add 30–100 μg of mitochondrial protein to the chamber.
7. Close chamber (Figure 7)

**Figure 7:**
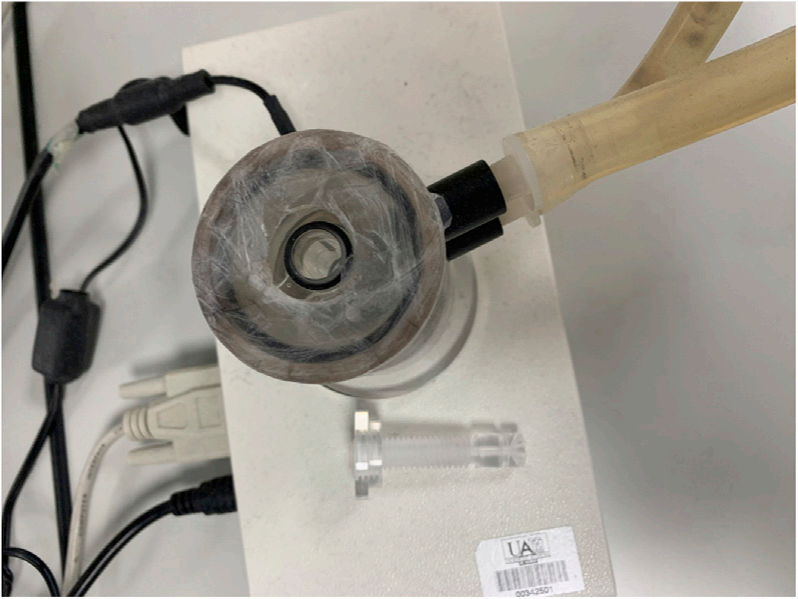
Clark-type electrode. Closed chamber is shown
8. Click *GO* to start recording the trace.

9. At 30 s inject one substrate and add a label (repeat it in each injection).
  ***Note:*** All injections should be performed with a Hamilton syringe to avoid opening the working chamber.
  a. 0.5 mM malate + 50 μM palmitoyl-carnitine: 5 μL from a 50 mM malate stock + 5 μL from a 5 mM palmitoyl-carnitine stock.
  b. 10 mM glutamate/malate: 10 μL from a 500 mM glutamate/malate stock.
  c. 10 mM succinate: 10 μL from a 500 mM succinate stock
    **CRITICAL:** using substrates in c. add 1 μM rotenone (5 μL from a 100 μM rotenone stock) before substrate injection to avoid retrograde electron transfer (RET) (Scialo et al., 2016). Alternatively, following ADP addition, pyruvate (final concentration: 25 mM) may be added to allow a measurement of flux control ratio of fatty acids relative to pyruvate (see (Horscroft et al., 2019)), followed by rotenone to consider RET.

10. At 2 min 30 s inject 250 μM ADP (5 μL from a 25 mM ADP stock).
11. Allow all ADP to be converted in ATP and slope returns as it with substrates only.

**CRITICAL:** Respiratory Control Index (slope in state III (P) / slope in state II (L) (at saturating [ADP] and [Pi])) for glutamate/malate should be > 4 for healthy controls (see below). For Skm healthy controls, values < 3 indicates poor mitochondria coupling.

**Figure 8:**
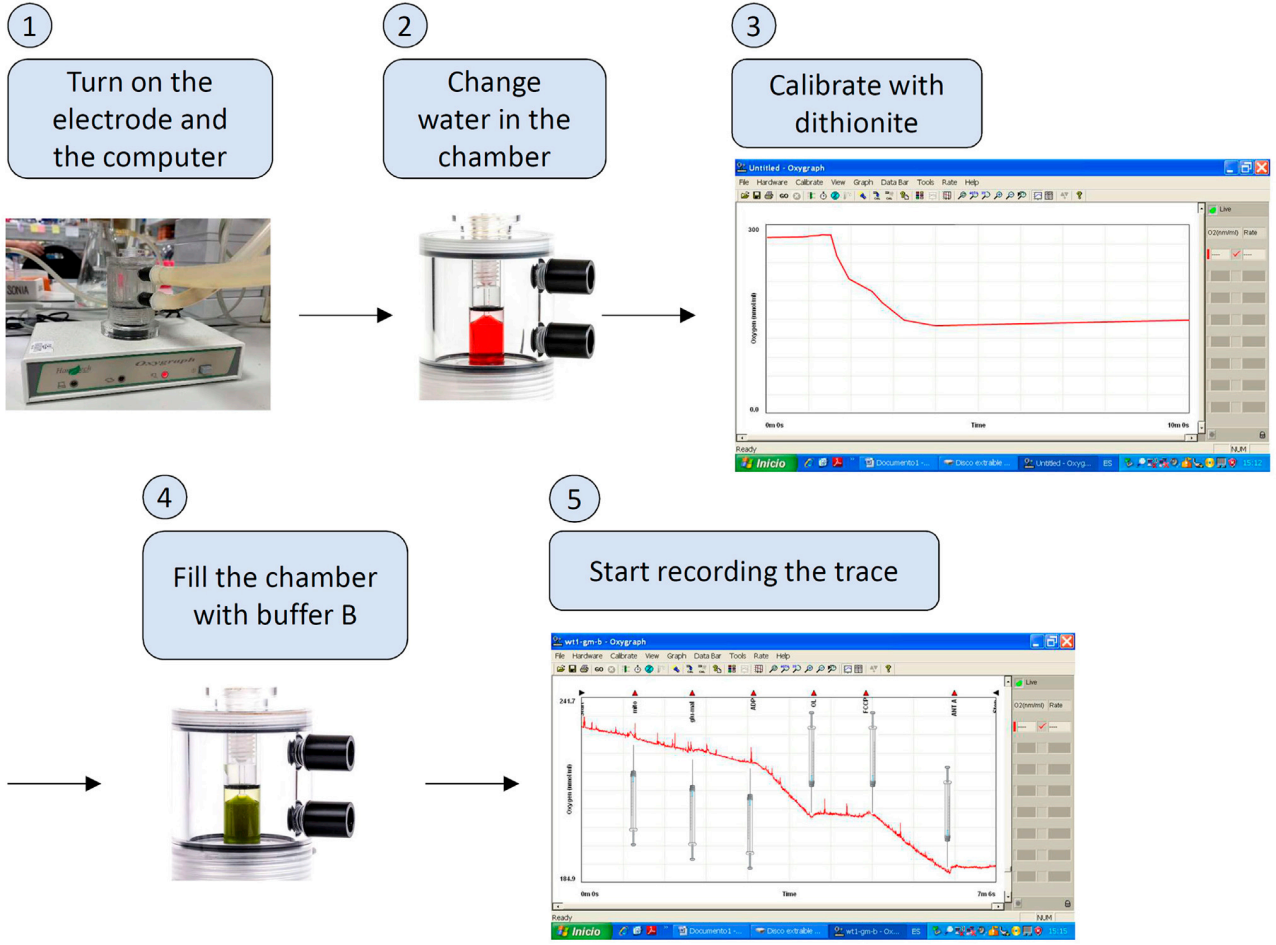
OCR measurement protocol

***Note:*** Allow sufficient time to permit the calculation of slopes in between injections. The time indicated here is flexible.

12. At 4 min 30 s inject 1 mM ADP (5 μL from a 100mM ADP stock).
13. At 5 min 30 s inject 5 μM oligomycin (5 μL from 500 μM oligomycin stock).
14. At 6 min 30 s inject 5 μM FCCP (5 μL from 500 μM FCCP stock)
15. At 7 min 30 s inject 1 μM antimycin A (5 μL from 100 μM antimycin A stock).
16. At 8 min 30 s stop measurement (click STOP button ) and save trace (click file, save as). A summary for OCR measurement steps is provided in Figure 8.

17. Repeat steps 5–16 at least 3 times for each substrate.

**CRITICAL:** Consider a 10% of initial oxygen concentration limiting for proper measurement. Ensure to stop the experiment before oxygen becomes limiting.

**CRITICAL:** carefully clean Hamilton syringe twice with EtOH and H_2_O after each injection.

## Expected outcomes

**Figure 9:**
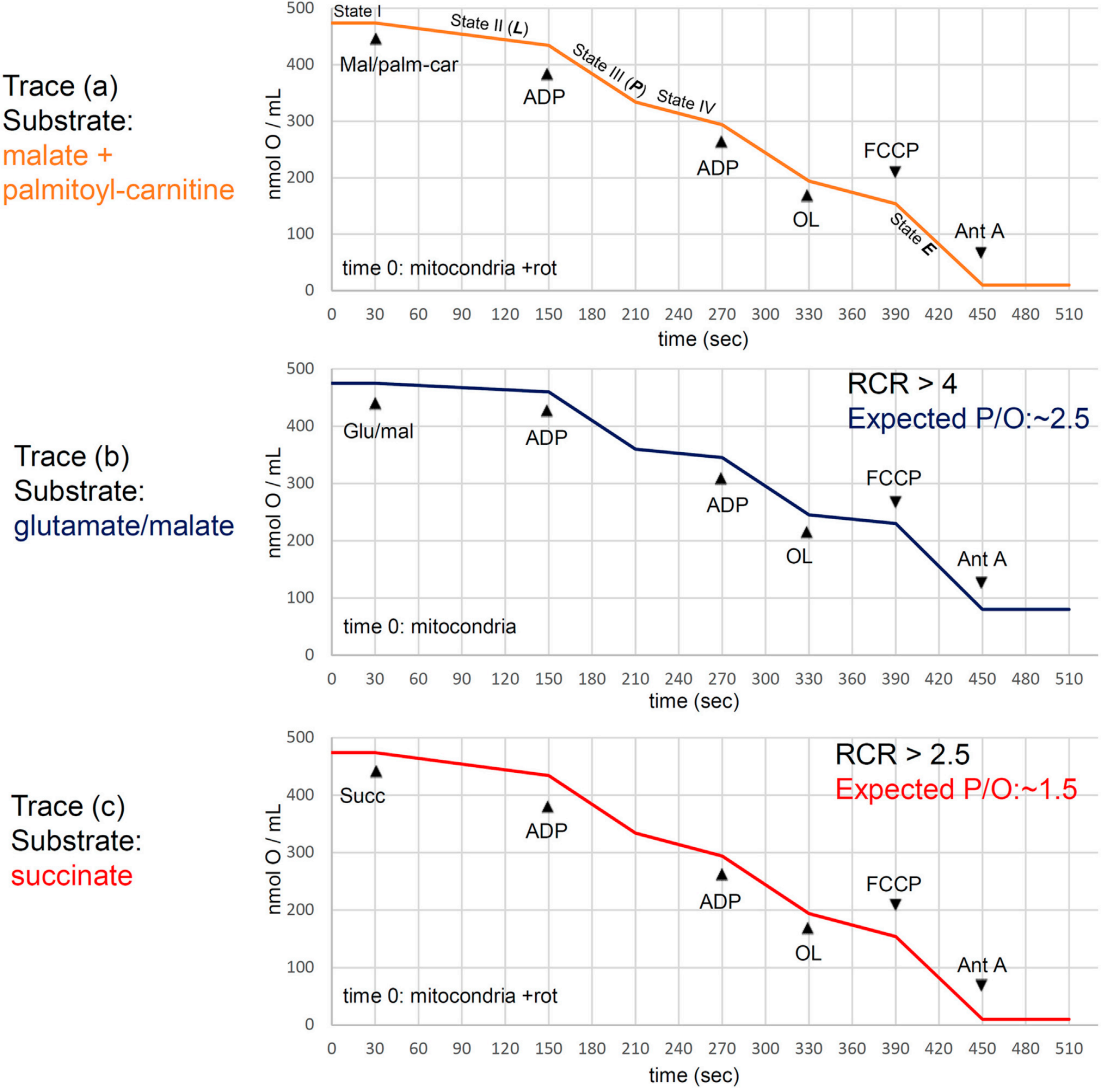
Expected traces in healthy mitochondria control

Expected control mitochondria trace using the Oxygraph+ system is presented in Figure 9.

## Quantification and statistical analysis

Adapted from (Nicholls, 2013) and (Gnaiger, 2020):

18. **Oxygen Consumption Rate** (OCR) is obtained by calculating the slopes of the different mitochondrial coupling states in a sufficient number of traces performed in the same conditions:
  a. State I: no energized mitochondria (consuming endogenous substrates until depletion, no ΔΨm).
  b. Leak State II (***L***): Resting, non-phosphorylating electron transfer with a short circuit of the H^+^ cycle across the mitochondrial inner membrane. It is a leak state in the absence of ADP and ATP. ETC substrates allow ΔΨm generation (maximum protonmotive force).
    ***Note:*** low respiration is expected due to the lack of OXPHOS.
  c. ADP-stimulated State III (OXPHOS capacity,***P***): ETC respiration is coupled with ATP production, allowing OXPHOS.
  d. d.1 Leak State IV (***L***): all ADP is depleted by phosphorylation to ATP, respiration drops (same slope that Leak State II). It is an ADP-limited resting state in the presence of ATP.
    d.2 Uncoupled State (ETC capacity, ***E***): uncoupled respiration with a short circuit of the H^+^ cycle across the mitochondrial inner membrane at optimum uncoupler (FCCP) concentration stimulating maximum O_2_ flux (maximum O_2_ consumption, very low protonmotive force).
    ***Note:*** A control-step includes the inhibition of ETC complexes to correct total O_2_ uptake for residual O_2_ consumption (Rox) (Gnaiger, 2020).
  e. State V: anoxia.
    ***Note:*** State III respiration corrected for Leak (P-L) is potentially available for net coupled phosphorylation of ADP to ATP (Gnaiger, 2020).

19. **Respiration Control Index/Rate** (RCI or RCR) is calculated for each substrate by dividing state III (P) slope per state II (L) slope (at saturating [ADP] and [Pi]). This value is a control index of coupling mitochondria. For Skm mitochondria, RCI for glutamate/malate should be > 4 in healthy controls (usually ∼5–6).
20. **Phosphate/Oxygen Ratio** (P/O) for malate + palmitoyl-carnitine (trace a in Figure 9) is calculated by dividing the generated ATP moles (= moles of ADP injected at step 10) per moles of consumed oxygen (O) (from the injection of ADP at step 10 until reaching the state IV). This value is representative of coupling ETC O_2_ consumption with FFA-β-oxidation.

***Note:*** P/O ratios of ∼2.5 and ∼1.5 with NADH-linked substrates or succinate, respectively, are expected.

**CRITICAL:** This value should be compared with P/O ratio for succinate (trace c in Figure 9) to discard late ETC dysfunctions in the sample mitochondria compared to control mitochondria.

Similarly, check the activity of the carnitine palmitoyltransferase I (CPT1), the enzyme responsible for FFA entrance to the mitochondria, to discard artefactual changes in trace (a) slopes. Radiometric (Dobrzyn et al., 2004);(Berthon et al., 1998), fluorometric (Schafer et al., 1993) and mass-spectrometry based (Sierra et al., 2008) assays have been described to measure it. CPT1 activity may also be investigated by respirometry using malate, palmitoyl-coA and free carnitine (See (Horscroft et al., 2019)).

***Optional:*** the day after the experiment, enzymatic activities of complexes I, II, III and IV (Santacatterina et al., 2016; Spinazzi et al., 2012) may be performed to discard single ETC complexes dysfunctions in sample mitochondria compared to control mitochondria.

***Note:*** these activities may also be investigated directly by respirometry using the following protocol:

Glutamate/malate; ADP; cyt c; succinate; FCCP; rotenone; antimycin A; TMPD/ascorbate; azide. This allows rates for coupled complex I (L+P), complexes I+II (P), complex II (P), residual oxygen consumption (Rox, non-mitochondrial), and complex IV (P) (Gnaiger, 2020)

21. **Oligomycin Sensitive Respiration** (OSR) is calculating comparing the state III slope with the slope obtained after the oligomycin injection. This value is representative of the H^+^-ATP synthase activity (Esparza-Moltó and Cuezva, 2018; Sanchez-Gonzalez et al., 2020).

***Note:*** H^+^-ATP synthase dysfunctions may result in alterations in ΔΨm (Formentini et al., 2012)

22. **Maximal Respiration** is calculating comparing FCCP-induced respiration with state 1 respiration
23. Statistical analyses are performed using a two-tailed Student’s t test. ANOVA and the Tukey's post hoc test is used for multiple comparisons, employing SPSS 17.0 and GraphPad Prism7 software packages. Bonferroni correction may be applied to avoid multiple comparison errors. p<0.05 is considered statistically significant.

## Limitations

At least one Clark-type electrode is required. However, note that Oroboros Oxygraph-2k may also be used to measure oxygen consumption.

Uncoupled mitochondria (or stored mitochondria preparations) do not work in Clark-type electrode, thus same-day mitochondria isolation and oxygen consumption assessment is mandatory.

**CRITICAL:** mitochondria tend to uncouple over time: use them immediately after isolation.

Coupled mitochondria may be difficult to obtain due to several limitations:

a. WAT deposits in *soleus* muscle (or any tissue) decrease mitochondrial coupling and increase proton leak (see Troubleshooting section).
b. Extra force or potter passes in the extraction decrease mitochondrial coupling (see Troubleshooting section).
c. Temperature is a critical feature of the protocol: mitochondria isolation must be performed on ice, pre-cooling all the material at 4°C, including buffers, centrifuges and potters.

Clark-type electrode experiments must be performed at constant temperature because O_2_ saturation level in media depend on temperature (see above). Electrode needs to be properly calibrated prior to OCR measurements.

Substrates and inhibitors might be degraded in freeze/thawing cycles; thus, it is strongly recommended to use compounds freshly prepared.

## Troubleshooting

### Problem 1

How to be sure that electrode is working properly after the manual set up (related to “Before you begin-*Clark-type Electrode preparation”* section, steps 10–13).

### Potential solution

Verification:
  Start recording. For Oxygraph+ program: Click *GO.*
  Wait for the trace to start.
  Stop: click *Stirrer OFF.*
  Wait for the trace to go down.
  Click *Stirrer ON.*
  Wait for the trace to restore initial level.

***Note:*** If no decrease in O_2_ (or only a slight decrease) is observed, the electrode does not work properly.

Solution:
  Dismantle the electrode.
  Clean the electrode with H_2_O milli-Q water.
  Add a drop of a saturated KCl solution in the platinum electrode.
  Put a new Teflon membrane.
  Put correctly the O ring (Figure 3, left panel) adjusting the membrane and avoiding bubbles.

### Problem 2

How to check if mitochondria are uncoupled (related to “Step-by-step method details-Skeletal muscle mitochondria isolation” section, step 4).

### Potential solution

This can be checked respirometrically by the addition of exogenous cytochrome c (cyt c, 10 μM) to mitochondrial preparations. If mitochondria have become damaged or uncoupled during the preparation step, the addition of cyt c will increase the oxygen flux highlighting the damage of the outer membrane and loss of the endogenous cyt c. See: (Pesta and Gnaiger, 2012)

### Problem 3

Mitochondria are uncoupled (low RCR) (related to “Step-by-step method details-Skeletal muscle mitochondria isolation” section, step 4).

### Potential solution

A wrong buffer composition/storage may affect mitochondria coupling.

Re-do all the buffers following right indications.

A too strong process of purification may uncouple mitochondria. Change to milder homogenization.
  Homogenize in a glass-glass homogenizer:
  8 times with potter A (smoother).
  8 times with potter B (stronger).
    ***Note:*** In certain cases, it could be recommended to perform only 1 centrifugation for nuclei separation and 1 for mitochondria isolation. Reducing the number of centrifugations reduces the purity of the preparation but increases mitochondrial coupling.
  Although fundamental interaction between lipid-storages and mitochondria has been recently described (Benador et al., 2019), the presence of excessive intramuscular adipocyte accumulation may result in excess of lipids during the isolation, thus altering permeability and uncoupling mitochondria (Rial et al., 1983). Carefully eliminate lipid phase with the help of a cotton swab after the first centrifugation (step 4f) to reduce lipid amount into the preparation.
  Increase the percentage of BSA in the buffer B.
    ***Note:*** BSA will bind lipids reducing their concentration.

Obesity and aging decrease RCR, increase mitochondrial proton leaks and alter mitochondrial inner membrane lipid composition (Cadenas, 2018; Formentini et al., 2017a; Sanchez-Gonzalez et al., 2020). Make sure that control animals are lean and young (2–8 months old).

### Problem 4

Mitochondria do not respond properly to substrates/inhibitors after few traces (related to “Step-by-step method details- OCR measurement by Clark-type Electrode” section, steps 5–16).

### Potential solution

This could be due to the presence of traces of inhibitors or impurities in the working chamber (bad or difficult cleaning).

Clean the electrode working chamber with 2 mL milli-Q H_2_O,2 mL EtOH, 2 mL milli-Q H_2_O.
Add 2 mL of PBS + 1% BSA to the working chamber for enhancing the clean effectiveness. Impurities and inhibitors will bind BSA.
Repeat steps 1 and 2 twice.

***Note:*** increasing the time of the washes also helps in removing inhibitors. Wash the chamber for at least 15 min in 100% EtOH between runs.

### Problem 5

Electrode is not sensitive (slow slops, instable traces) (related to “Step-by-step method details- OCR measurement by Clark-type Electrode” section, steps 5–16)

### Potential solution

A well-known cause of failures of oxygen sensors is the appearance of gas bubbles. The unequal rates of the heating of the measuring system’s components are the most probable (but not unique) reason of the diffusive flow of oxygen through the membrane of the sensor:
  Make sure heating system works properly and constant temperature is maintained
  Adjust stirring and avoid any vortex, which can change the reading by adding oxygen from the ambient air.
  Eliminate gas bubbles in the proximity of the Teflon membrane (Figure 2)
Consider increasing the concentration of mitochondrial protein in the chamber. Mitochondrial activity decreases with aging (Kauppila et al., 2017) or pathologies (Balaban et al., 2005; Dillin et al., 2002; Formentini et al., 2012; Formentini et al., 2017b; Nuevo-Tapioles et al., 2020)

## Resource availability

### Lead contact

Further information and requests for resources and reagents should be directed to and will be fulfilled by the lead contact, Laura Formentini (lformentini@cbm.csic.es).

### Materials availability

This study does not need any new reagents.

## Data Availability

This study does not generate/analyze data sets or code.
